# New Hope for a “Cursed” Crop? Understanding Stakeholder Attitudes to Plant Molecular Farming With Modified Tobacco in Europe

**DOI:** 10.3389/fpls.2020.00791

**Published:** 2020-06-12

**Authors:** Jonathan Menary, Mario Amato, Andrés Cid Sanchez, Matthew Hobbs, Agata Pacho, Sebastian S. Fuller

**Affiliations:** ^1^Institute for Infection and Immunity, St George’s, University of London, London, United Kingdom; ^2^Department of Political Science, University of Naples Federico II, Naples, Italy; ^3^Department of Microbiology, Centro Technológico Agroalimentario Extremadura (CTAEX), Badajoz, Spain

**Keywords:** plant molecular farming, pharming, new plant breeding techniques, qualitative research, responsible research and innovation, tobacco

## Abstract

Plant molecular farming (PMF) with tobacco could provide a sustainable and cheap platform for the production of high-value proteins for medical use. It could also offer European tobacco farmers an alternative, healthful end use for their crop. New plant breeding techniques (NPBTs) offer a means of quickly and precisely optimizing molecular farming platforms for this purpose. However, there has been little empirical research focussing on the barriers and facilitators of these technologies in the agricultural sphere. Here, we explore key stakeholder perceptions toward this combination of technologies, exploring their understanding of risk and opportunity. We interviewed *N* = 24 key stakeholders – tobacco farmers, agronomists, policymakers, and researchers – in three tobacco-growing areas of Spain and Italy. Our findings demonstrate these stakeholders have a favorable attitude toward PMF with tobacco due to its beneficial medical purpose and the opportunity it provides farmers to continue growing tobacco in a declining European market. Tobacco producers also reported favorable views toward NPBTs, though for some this was contingent on their use for non-food crops like tobacco. Most stakeholders’ concerns are economic in nature, such as potential profitability and demands for new agronomic practices or infrastructure. Tobacco producer associations were thought to be important facilitators for future PMF scale-up. The attitude toward these technologies by smoking tobacco companies is, however, unknown and constitutes a potential risk to the development of PMF.

## Introduction

Plant molecular farming (PMF) produces high-value molecules in plants. These include proteins for medical use (Ma et al., 2005, 2015; Teh et al., 2014; van Dolleweerd et al., 2014; Budzianowski, 2015; De Martinis et al., 2016; Dhama et al., 2018) and secondary metabolites for a variety of uses, such as industrial enzymes and cosmetic products (Tschofen et al., 2016). Demand for these molecules has grown and continues to rise, sometimes outstripping supply from existing protein expression systems that rely on yeast, bacterial, and mammalian cell cultures (Obembe et al., 2011; van Dolleweerd et al., 2014). Plants are an attractive alternative to these expression systems due to their potential for: (i) lower up-front production costs; (ii) low risk of contamination with human pathogens; (iii) scalability of cultivation; and (iv) expertise and infrastructure in place for the production of plant material (Horn et al., 2004; Twyman et al., 2005; Ma et al., 2013; Xu and Zhang, 2014; Tschofen et al., 2016). It has been noted that the scale-up of PMF could represent a significant boost to global health (Paul et al., 2011; Buyel et al., 2017). Currently, PMF research is being carried out in North and South America, Europe, Japan, China, Thailand, Australia, and South Africa (De Martinis et al., 2016; Murad et al., 2020).

PMF represents the “third” generation of biotechnology (Sakakibara and Saito, 2006) and new plant breeding techniques (NPBTs) – a suite of technologies that allow the precise modification of plant germlines – can improve PMF platforms for better expression of target proteins (Mercx et al., 2016). NPBTs include site-specific nucleases, such as CRISPR Cas-9, which can induce precise, single base-pair modifications to plant genomes. Whilst certain biopharmaceutical PMF involve the use of transgenes, “gene editing” makes possible vertical cisgenic or intragenic changes in plant genomes (i.e., using only the “native” genetics of a plant in natural or novel combinations). NPBTs have renewed the debate on how best to regulate plant breeding technology, particularly in Europe where public reaction to genetically modified (GM) crops has been both pronounced and instrumental in shaping biotechnological trajectories (Ma et al., 2005; Hartley et al., 2016; Malyska et al., 2016; Tanaka, 2017; Pei and Schmidt, 2019). Current European Union (EU) regulation dictates that plants bred through the use of NPBTs are considered GM crops, even if the resultant plant line contains no transgenes (Eckerstorfer et al., 2019). The 2018 decision of the European Court of Justice on this matter has caused concern amongst scientists over the future of European biotechnology investment and the ability of existing systems to effectively monitor such organisms (Wight, 2018; Ledford, 2019). The risks of biotechnology have often been understood in terms of potential environmental or human health hazards; yet risk must be weighed against opportunity, which can be defined as the potential benefits of a technology (Raybould and Macdonald, 2018). However, what constitutes risk and opportunity is not always clear. Some authors have argued that perceptions of risk are shaped by local social factors and that these social understandings of risk must be explored for a true accounting of technological risk (Guehlstorf, 2008; Pavone et al., 2011; Binimelis and Myhr, 2016). NPBTs (and PMF) could change our understanding of the ethical implications, risk and opportunity of biotechnology due to its potential to confer consumer or social benefit (Nevitt et al., 2006), whilst being closer to the conventional breeding practices farmers have traditionally employed (Malyska et al., 2016). Some authors have also noted that understanding farmers’ opinions toward biotechnology could provide a “middle ground” between advocates of biotechnology and its opponents, arguing that because farmers will continue to face choices about biotechnology policy planners should have an understanding of the demand for GM crops amongst key stakeholder groups (Guehlstorf, 2008).

The use of crop plants for PMF bridges both the agricultural and health sciences, which have distinct processes of implementing research into practice (Menary, 2015). When it comes to understanding the adoption of agricultural technology or practices, studies range in scope from the local and specific (D’Emden et al., 2008; Howell et al., 2015; Aryal et al., 2018), to the broad and general (Pierpaoli et al., 2013; Kuehne et al., 2017). Some focus on behavioral characteristics of farmers in the adoption of new practices (Dessart et al., 2019) and others on characteristics of the practice or technology itself (Kuehne et al., 2017). These studies often rely on quantitative methodologies, particularly surveys and econometric approaches, in order to identify patterns of adoption and make predicative models for the spread of new agricultural technologies.

Rather than see farmers as passive adopters of biotechnology, though, other frameworks see them as vital components of the innovation process itself. The agricultural innovation systems framework (AIS) emphasizes the importance interaction amongst farmers, researchers, agronomists, input suppliers, and policymakers for innovation (Rajalahti et al., 2008; Klerkx et al., 2010). These interactions are in turn shaped by relevant institutional and policy environments, which often require social innovation to permit technological change or improved agricultural practices (Turner et al., 2017). Howell et al. (2015), for example, demonstrate that farmers in Nepal were unlikely to adopt improved water management techniques due to local institutional factors. Innovation is therefore often cast as the co-evolution of social and technological systems (Schut et al., 2015).

Recent literature has also explored “transitions,” that is, the wider changes in the socio-technical regime as society and technology develop (Geels, 2010). The multi-level perspective describes a number of levels – niches (where radical innovation emerges), the socio-technical regime (current ways of doing things that are stabilized through behavior, vested interests and existing regulations) and the socio-technical landscape (the slow-changing exogenous environment) – that can be used to explain the gradual development of PMF in Europe. Menary et al. (2020) describe how the existing socio-technical regime has been one of several factors constraining PMF; existing regulations do not reflect the advantages offered by plants and significant investment in cell culture alternatives by pharmaceutical companies deters investment in plant-made alternatives.

For these systems-based approaches it is necessary to understand local conditions, policies and issues for key stakeholders. We argue that this is no less true for open-field PMF. A number of studies have focussed on such contexts, predominantly in United States tobacco-growing regions, where since the early 2000s the idea of substituting traditional smoking tobacco for PMF tobacco has gained attention (Nevitt et al., 2006, 2003; Hayes et al., 2014). These studies concern first-generation, transgenic PMF tobacco; none have yet explored the development cisgenic or intragenic PMF tobacco, for example, nor the prospect of PMF in Europe. As such, there is a need to explore the perceptions of risk and opportunity amongst key stakeholders and understand the potential barriers and facilitators for the responsible scale-up of PMF. Recent studies have also highlighted the need for consultations with producers and end-users in the development and governance of science and innovation, such as called for under the Responsible Research and Innovation (RRI) framework, the principles of which are encompassed in the Horizon 2020 programme (Stilgoe et al., 2013; Loureiro and Conceição, 2019). There is a need to understand how the biotechnology that makes PMF possible will be perceived by its potential stakeholders and how this technology could be implemented in the European context.

The Newcotiana project^1^ is funded by the European Commission Horizon 2020 programme and led by Consejo Superior de Investigaciones Científicas (CSIC) in Spain, involving 18 institutions in EU member states and one in Australia. The programme of work focuses on the application of NPBTs to improving the tobacco plant (*N. tabacum)* and a closely related cousin (*N. benthamiana)* as PMF platforms. The tobacco plant has been used as a model crop for a number of decades and became the first transgenic plant in the early 1980s. *N. benthamiana* is used for similar purposes and has recently been used in the transient (i.e., temporary) expression of antibodies for HIV treatment (Lombardi et al., 2009; Teh et al., 2014). These plants have been described as the “white mice” of the plant kingdom (Nevitt et al., 2003). Alongside product-focused targets, generic crop improvements, such as various stress tolerances and the suppression of flowering are also planned. Non-flowering traits are a biosafety feature intended to prevent outcrossing with other crops. Coupled with stagnant or declining tobacco production in some EU member states (European Commission, 2015), PMF tobacco could also offer producers a new and more profitable end use for their crop. The gradual substitution of tobacco for other crops has also been promoted through reforms to the Common Agricultural Policy (CAP) in recent years (Geist et al., 2009). Likewise, the EU’s “Bioeconomy Strategy” promotes the development of “greener,” more sustainable and circular industrial processes (European Commission, 2018). PMF could represent a valuable contribution to those aims.

Important questions remain over the reliability and safety of plant-derived molecules, particularly where these are intended for medical use (Ma et al., 2015). Open-field PMF presents a number of additional challenges in this respect, such as the uniformity of plants grown under changeable cultivation conditions and the risk of contamination, both in terms of the molecules themselves and the unwanted spread of GM plants (Mascia and Flavell, 2004; Breyer et al., 2009). Whether open-field PMF for biopharmaceuticals can ever meet the strict criteria laid out in good manufacturing practice guidelines has been questioned by leading PMF scientists (Menary et al., 2020). However, the development of enriched, open-field Newcotiana *N. tabacum* feedstock lines that are intragenic (i.e., containing novel combinations of native genetics but no transgenes) and destined for biorefinery use is intended to be a “proving ground” for the efficacy of NPBTs in Europe and a means to demonstrate the reliability of PMF platforms.

Here we present the findings from our interviews with key stakeholders in the tobacco farming industry on their opinions toward PMF and NPBTs.

## Materials and Methods

Our study employed semi-structured interviews to generate qualitative data (62), which were chosen to probe perceptions of risk and opportunity around new technology and the systemic factors that influence the tobacco supply chain as it exists today and could exist for PMF tobacco.

Following previous research that explored the perceptions of Newcotiana consortium researchers and businesses toward the barriers and facilitators of PMF and NPBTs (Menary et al., 2020), relevant key stakeholders in the production and distribution of tobacco were hypothesized to be existing tobacco farmers, their advisors and producer associations, as well as policy makers and researchers familiar with tobacco production. Onward supply chain stakeholders such as pharmaceutical companies and regulators are being queried in other Newcotiana work packages.

Our selection criteria for study sites were: (1) sites should be in countries with large-scale tobacco production in the EU and (2) study sites should represent major tobacco growing areas within those countries.

An interview guide was designed to probe: (i) participants’ involvement in tobacco production, (ii) challenges for modern tobacco production, (iii) participants’ attitudes toward new end uses for tobacco, (iv) perceptions of genetic modification and NPBTs, (v) and potential barriers to up-scaling PMF in their communities and countries (see Supplementary Material). The participant information sheet detailed the aims of the project and the reason for participants’ involvement. The information sheet was available in the predominant local language and consent was given by all participants in advance of the interviews. Interviews were conducted by JM, a PhD-level social scientist with 6 years of experience in qualitative research. Additionally, to ensure we could interview participants in the language they were most comfortable, we employed a quasi-peer interviewer technique in which researchers MA (Italian) and AS (Spanish) facilitated interviews alongside JM. MA is a research fellow at University of Naples Federico II and AS is a biologist with Newcotiana partner institution CTAEX. No relationships existed between interviewer(s) and participants prior to the study. However, gatekeepers (contacts at tobacco producer associations) purposively selected people who they knew to be members of the professional tobacco growing community in that area to invite to interview.

### Data Analysis

Interviews were fully translated and transcribed into English using a professional translation and transcription service and were then checked for accuracy by the relevant interviewers (JM, MA, and AS). Any identifying information of participants was removed. The transcripts were uploaded into NVivo 12 for data management and analysis.

Data analysis was undertaken in accordance with *Framework Analysis*, a thematic approach for large-scale policy work (Ritchie and Lewis, 2010). It is designed for instances in which there are specific questions, a pre-designated sample (often professionals in a given domain), limited timeframes and known *a priori* issues (Srivastava and Thomson, 2009). An initial coding framework was developed for the interviews conducted in Italy by JM, SSF, MH, and AP. SSF and AP are both PhD-level social scientists with >10 years’ experience with qualitative data collection and analysis; MH is a social science research assistant with 2 years’ of experience. The researchers then checked the Spanish interview transcripts against this coding framework, judging as a group many of the same themes to be present in both datasets with minimal need for refinement. Subsequent indexing was undertaken by JM using NVivo 12.

St George’s University of London ethical review committee gave approval for this study (reference: SGREC18.0006).

## Findings

**FIGURE 1:**
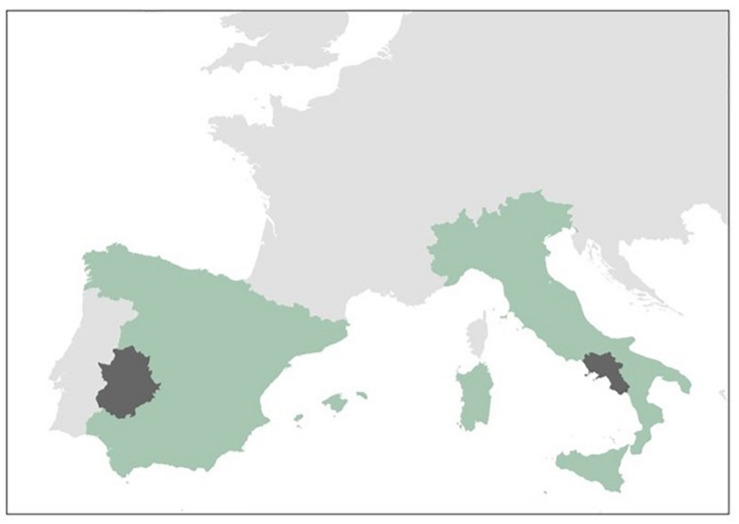
A map of Western Europe showing Extremadura (Spain) and Campania (Italy).

Four countries were considered for inclusion: Greece, Italy, Poland, and Spain. Contact was made with gatekeepers (researchers and tobacco producer associations) in these areas, with Italy (Campania) and Spain (Extremadura) being chosen as suitable study locations on the basis that our study site selection criteria were met and that interviews could be arranged with a variety of individuals associated with tobacco production (see Figure 1). Spain and Italy together represent ∼40% of EU tobacco production (63).

In total, *N* = 24 interviews were undertaken at three sites (two in Campania and one in Spain) between November 2018 and February 2019 – 15 were with tobacco producers, seven with tobacco technicians^2^, one with a policy expert and one with a researcher specializing in (tobacco) agricultural engineering. Five participants represented two categories, the most common pairing being farmer and technician. The majority of participants (*n* = 20) were male. Interviews lasted between 25 and 55 min and took place in the offices of the local producer associations. Interviews were recorded by Dictaphone and field notes were made by the research assistant (JM) during and after the meetings. MA and AS helped organize the meetings, translate questions and answers, review interview transcripts and the initial analysis. A unique participant number is used following quotations used below.

### Tobacco Production in Campania and Extremadura

Tobacco was first introduced to Italy in the sixteenth Century. Today, Italy is the EU’s largest producer of tobacco. Over 50,000 tons are produced each year, the primary locations for tobacco production being Veneto, Umbria, Tuscany, and Campania (Martino et al., 2014). Campania is a region in southern Italy. Most tobacco is grown to the north on a flat plain around Caserta and north-east in the Apennine area near the town of Benevento. The only variety of tobacco grown in Campania is Burley (“Kentucky”) tobacco, which is used in “Toscano” cigars (Raimo et al., 2016).

The average tobacco farm in Campania represented in the present sample (*n* = 8) is 7.3 ha, the smallest being 4 ha and the largest being 10 ha (with a standard deviation of 1.8 ha). These were self-reported figures of tobacco-growing area only. It should also be noted that many tobacco producers have other holdings, but reserve land for cereals (primarily wheat), olives, and vegetables. Most tobacco farms in the area are family farms organized as independent businesses that are affiliated to one of several producer associations in the area. These organizations facilitate several activities on behalf of tobacco farmers^3^, such as contract negotiation, providing loans for infrastructure and machinery and agronomic advice throughout the growing cycle. Approved seed is distributed either through producer associations or through local producers under license [several of the participants in the study grew seed on behalf of Philip Morris International (PMI)]. Seed is raised under cover in polytunnels or glasshouses before being transplanted to the field. A team of workers (sometimes including the farmer’s family) harvest the tobacco leaves three to four times between July and early September; the hand-picked leaves are strung together then hung to (air) dry before being manually sorted and boxed.

Industrial-scale tobacco production began in Extremadura following the “internal colonization” policies of the Franco era, which established rural communities in newly irrigated dry regions (Naylon, 1967). Today, Spain produces 32,000 tons of tobacco, predominantly in the region of Cáceres, close to the *Tiétar* River and at the foot of the *Sierra de Gredos*. The Virginia (bright-leafed) variety of tobacco is grown in Extremadura at scales considerably larger than in Campania, as this crop can be mechanically harvested. The average size of tobacco farms in Extremadura represented in the present sample (*n* = 8) is 46.4 ha, the smallest being 10 ha and the largest 92 ha (with a standard deviation of 34 ha). These were self-reported figures of tobacco-growing area only. As in Campania, most tobacco producers also reserved sizable portions of their land for other crops, including cereals (primarily maize), soft fruit and chili peppers. Most farms are family owned but affiliated with one of three large producer associations in the area. These associations, however, also manage communal flue drying facilities (available for use among association members), as Virginia tobacco is dried in bulk. The associations sort and box the tobacco on behalf of their members before sale to Deltafina, a subsidiary of Universal Corporation, which supplies Altria and PMI.

### Current Challenges in Tobacco Production

Our participants reported a range of challenges for tobacco production in their respective countries, the most significant being stagnant prices paid for the crop and rising costs of production. In Spain, producers claimed that due to CAP reform, tobacco had become unprofitable and is sold at below market rates – yet government subsidies have meant that tobacco production can continue. Many producers have turned to other crops to supplement their income (though one participant had recently given up on tobacco altogether, citing falling profitability as the primary reason).

A key concern for producers is the availability and proficiency of farm workers, who have become harder to find in recent years due to the physical difficulty of the work involved and reliance on a diminishing number of migrant workers. As one Spanish producer noted: “Until this year, we have worked with foreign workers. But already this year we had some difficulties… you don’t find foreign workers anymore” (Participant 16).

The producers also spoke of the social stigma associated with tobacco, one declaring that “the crop is cursed.” Although many described feeling stigmatized, none had experienced an outward manifestation of stigma from members of the public. Participants described feeling that campaigns against smoking were a challenge to their livelihoods: “… because there are worldwide anti-smoking campaigns, you are affected, even as a producer” (Participant 24). Some were more defensive, arguing that it remains legal to produce tobacco (they are “not producing smokers” or “killing anybody,” after all) and that farmers of other crops, such as wine grapes, are rarely accused of producing “alcoholics” in the same way tobacco producers are associated with the users of their product. A participant with significant holdings in Spain called for tobacco production to be banned outright rather than be “strangled” by low prices and dependence on subsidy (they did not see themselves abandoning tobacco for any other reason). These views point to a strong sense of identity as tobacco producers, caught between their traditional way of life (many were second- or third-generation tobacco producers farming family land), social pressure and difficult economic circumstances.

The impacts of climate change were cited as a significant problem for producers and technicians. Several participants described more extreme weather: “I remember when I was a kid I used to go to the fields and there was a lot of dew, wetting your shoes until 10, 11 in the morning. Now it’s like the desert” (Participant 19). Other environmental factors, such as pests, were also a source of concern. The main pests (according to participants) are tobacco flea beetle (*Epitrix hirtipennis*), nematodes and downy mildew (*Peronospora*). A variety of phytosanitary measures exist for the control of these pests, but the withdrawal of particular active ingredients in recent years within the EU has led to less effective pest control: “… in terms of plagues and diseases, many chemical products are disappearing, many active [ingredients] are being regulated. So we have to search for other means of controlling those” (Participant 2). In order to overcome the challenge of shrinking chemical portfolios, technicians spoke of collaborating with local agricultural administrations and private companies to explore alternative pest control options. Experimentation was a recurrent theme amongst producers and technicians alike: “I have already cooperated in the past with the University of Naples for studies on phytosanitary products” (Participant 15). Another producer described devoting a two-and-a-half hectare area of his growing area to trialing different varieties of tobacco.

### New End Uses for Tobacco

As described above, tobacco producers saw themselves as the farmers of a crop and not, as stigma around tobacco might suggest, the growers of illicit substance. The end-use of their crop did not personally concern them in most cases, though the prospect of a more healthful purpose for tobacco was, conversely, described in strong terms: “… it would be like meeting the Messiah, if tobacco could save lives” (Participant 1). There was a recognition that PMF could provide a more economically sustainable future for tobacco as the demand for cigarettes “decreased.” Some participants also expressed a desire to continue growing tobacco: “… anything that helps us continue doing what we have done all our lives, would be fabulous” (Participant 6).

There was also a perception that PMF tobacco could improve the image of tobacco producers in the eyes of the public, thus challenging the social stigma they felt. However, discussion centered on the potential economic returns of PMF: “It is important there is remuneration, an economic return…” (Participant 22). Likewise, technicians expressed concern over what new agronomic demands the crop might have and what new infrastructure might be required – the regulatory status of PMF crops was also questioned, with many participants familiar with current EU legislation on biotechnology and reported the fact that the genetic modification of tobacco had been resisted by “Big Tobacco” in the past (see below). Some producers were dismissive of hypothetical questions about PMF, expressing a desire to instead work directly with new tobacco varieties: “There is no sense talking about it now. If they decide to start, we’ll do a trial … then we can talk directly to each other” (Participant 16).

### Attitudes Toward NPBTs

In general, participants were not opposed to the use of NPBTs – described to participants as genetic modification under current EU law – and some producers in Spain had elected to grow GM maize. For those expressing concerns about genetic modification, perceptions of risk differed between PMF and other GM crops: “I mean, as a grower we have always been against [GM]. But in this case, you understand, it is different. The product is different” (Participant 16). A producer association representative summarized the distinction as follows:

*“… GM scares a bit, but what is the reason of this fear? For its effect on human health. I mean, when you eat something, yes, a food produced from GM plants, so you are worried about the effect on your health. When the product is simply used to extract some molecules… it doesn’t have a negative impact on that”* (Participant 18).

The distinction between food and non-food crops is significant, but approval also rested on the *purpose* of PMF as a beneficial alternative to smoking tobacco: “… something that, with a gene change can improve health? I’d be delighted” (Participant 5).

Most participants suspected the beneficial purpose of PMF would lead to NPBTs being seen as more socially acceptable: “If the product has a good purpose, why not?” (Participant 3). One, however, used a local phrase (*“ogni capa è ‘nu tribunale”* or “every head is a court”) to cast doubt on the extent to which public reaction can be predicted. It was noted by some participants that transgenic tobacco is banned in the EU.

The distinction between NPBTs and first-generation, transgenic GM was discussed although some producers were dismissive of such “scientific aspects,” having no concerns with current GM crops or viewing the vertical gene transfer as being equivalent to conventional hybridization. No ethical concerns were raised, though one participant did claim that if animals were being modified through the use of biotechnology, it was by extension acceptable in plants. Several participants did see an opportunity for more general agronomic improvements in tobacco. The suppression of flowering was noted as being of particular importance, as one Spanish grower explained: “We [would] save 15, for sure 10 percent of the production costs… less nitrogen and phosphates that they say we pour into the rivers” (Participant 1).

### Existing Supply Chain Actors and EU Policy

Several elements of the existing systems that mediate modern European tobacco production were cited as being important for any new technological development in the sector. A high-degree of trust was placed in the operation of the producer associations (POs) – arranged as either businesses in their own right or as farmer cooperatives – to which tobacco producers must belong in order to maintain contracts with tobacco-buying firms. As one producer noted: “It is a good association, we believe in tobacco… and we hope to continue with this organization, because otherwise, it they weren’t here everything would have already stopped” (Participant 3). POs facilitate these contracts, as well as providing necessary loans, equipment and agronomic advice on varietal selection and pest management.

The “success” of PMF was considered to hinge on the support of POs. The important role they play in farmer learning processes is particularly relevant. Most producers received information about new developments in the tobacco sector through their PO (and technicians in particular): “Through [PO], we get lots of good information” (Participant 8). The internet, agricultural shows both at home and abroad and word of mouth were also cited as important in this respect. The interconnectedness of the European tobacco associations leads to the quick spread of information: “Anything, any innovation in the world of tobacco spills over to all other countries. And, of course, it’s a well-structured group. We belong to cooperatives, those groups have a professional body where all the processors and the producers and everything at the new European level is connected” (Participant 7).

There was a degree of uncertainty concerning the stance of “Big Tobacco” – large tobacco-buying firms such as PMI, the main buyer for most Spanish and Italian tobacco – toward the prospect of PMF. Several participants suggested that such firms could play a positive role in the development of the technology. Others were somewhat suspicious of businesses that, having considerable power in the tobacco supply chain, had opposed the use of certain biotechnologies for tobacco in the past, as one policy expert explained: “If they change their opinion (because they change their opinion a lot), you have to be able to adapt. Until now, they have not been in favor of this kind of thing. And when there were talks about genetically modifying tobacco, they never agreed” (Participant 4). The response of the European Commission to the prospect of modified PMF tobacco was also considered to be of paramount importance for its development.

The questions that participants had for us as representatives of the Newcotiana project are described in Box 1. These are important because they provide examples of the types of information that would be required for existing tobacco producers and others to consider adopting these technologies in future.

**Box 1. Selected questions generated by participants.**

• Is PMF profitable?
• Have PMF crops ever been tested in the field?
• Why is the research team focussing on Europe and not elsewhere?
• Are there any Italian partners involved in the project?
• Are there any plans for PMF crops to be introduced in Spain?
• Are green (i.e., non-dried) or dried leaves needed to produce the desired molecules?
• What quantities of tobacco are required?
• Will a new supply chain be required?
• What new infrastructure will be required?
• What technical progress has been made on the Newcotiana project?

## Discussion

As Ferrell (2012) suggests, telling the “true” story of tobacco farming is troublesome due to the stigma around the crop and those who grow it. Tobacco farmers can be wary of outsiders and outsiders themselves have strong, pre-formed opinions of the sector. The Newcotiana project brings to this stigmatized sector a likewise controversial form of plant breeding (biotechnology). However, the findings above demonstrate a number of perceived risks and opportunities around new technology. In the following section, these are discussed and placed within a framework of barriers and facilitators to the responsible scale-up of PMF in Europe.

### Substitution of Tobacco

**FIGURE 2:**
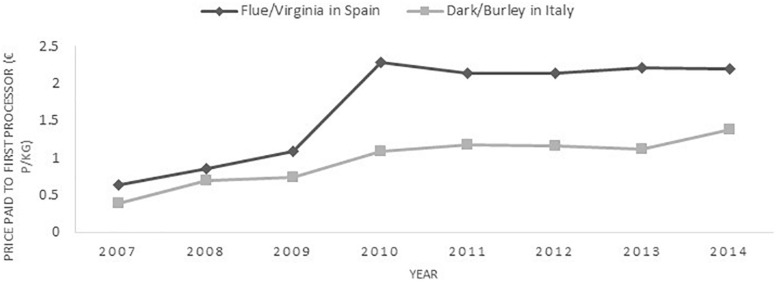
Price of tobacco paid by first processor between 2007–2014 in Spain (flue, Virginia) and Italy (dark, Burley). The chart does not include agricultural subsidies. Data derived from Raw Tobacco - Production Statistics - 2014 - 2003 Harvests (European Commission, 2015).

There are distinct parallels between the challenges faced by European tobacco farmers and those facing their counterparts in the United States: changing technologies, rising input costs, stagnant or falling prices, labor shortages and pressure to expand and/or diversify their operations (Ferrell, 2012). What data are available to support these claims corroborate the picture of challenging economic conditions for tobacco producers. The price paid for tobacco in Europe by first processor remained relatively stagnant between 2010 and 2014 at ∼€2 per kilogram (more recent data are yet to be published), suggesting that any increases in costs must be absorbed by cost-cutting measures elsewhere (see Figure 2). Stagnating prices may explain why producer concerns about PMF are predominantly economic (Nevitt et al., 2003). As noted earlier, the EU has encouraged tobacco substitution by decoupling subsidies from production (Geist et al., 2009), although the decisions of individual members states – as in Spain – have preserved tobacco cultivation even where this has become economically questionable. Our participants perceived PMF as a distinct opportunity to continue with tobacco and remain profitable longer-term. In a study of 145 tobacco farmers in five US states, economic incentives prove a key factor for the adoption of PMF tobacco (though increased returns are required to match requirements for new equipment and changes in production methods) (Hayes et al., 2014).

Finding workers was also cited as a problem by participants and, as Geist et al. (2009) show, this extends to northern Europe where recruitment and cost of labor pose significant problems. However, Italy and Spain report the highest dependence on non-family, non-regular agricultural workers in Europe (European Union, 2017). Although the importance of maintaining rural livelihoods is often invoked by tobacco-based firms as a reason to avoid further tobacco control (or the promotion of substitution for other crops) (Leppan et al., 2014), few studies have quantified the impact of substitution on rural livelihoods. Research in the Spanish context has found that labor requirements in Extremadura would fall between 14.1 and 42.8% in various tobacco substitution scenarios, demonstrating these concerns are at least partially justified (Kienle et al., 2015). PMF tobacco could bolster farm incomes providing a premium is guaranteed, but it could also decrease reliance on labor (depending on condition of leaf required and suppression of flowering time).

Our research suggests that small-scale, *partial* substitution of smoking tobacco for PMF tobacco is the most likely first step in any future transition. This aligns with existing research, in which most farmers saw PMF tobacco as opportunity to complement their existing crop portfolio, with only 15.4% willing to grow PMF tobacco on more than 31% of their acreages (Nevitt et al., 2003).

Not only would partial substitution satisfy a desire that many participants had to continue growing tobacco, there is also opportunity to make use of farmers’ existing knowledge and competencies. It would also represent tobacco production “parallel” to (but not necessarily intersecting) traditional tobacco farming (Nevitt et al., 2003). This would ensure existing commercial relationships continue – mitigating risk for producers – and preserving the structure and purpose of POs so that they can help facilitate the development of PMF (see below).

The producers we spoke with were confident in their abilities to produce a high-quality crop to specification. However, the expressed desire by participants to first trial new varieties of tobacco indicates that a period of experimentation will be required; some producers mentioned having an area of their land given over to such field trials and it would be likely that these would form the first test beds for any new varieties. Importantly, most farmers were not deterred by potential co-existence policies for PMF tobacco, such as fallow zones between fields and 1-year restrictions on growing non-PMF crops on land previously used for modified tobacco. Such co-existence policies are currently in force for the EU’s only approved GM crop varieties (maize), which specify that these crops must be isolated from non-GM cropping systems. Some form of isolation distance is expected for PMF tobacco (Elbehri, 2005), though Herrero et al. (2017) suggest that in Spain such measures are not enforced and have led to social conflict between farmers of different production systems.

Existing research on tobacco substitution has found a range of personal (farm-level) and systemic factors that can influence such transitions. Many of these are action research projects in low-income countries in which researchers are embedded in tobacco-growing communities – such a model may also be of interest for PMF researchers in the future. Akhter et al. (2014) employed an action research approach to instigate change in tobacco growing communities in Bangladesh. Their study found that supporting tobacco producers in diversifying their crop portfolio facilitated the transition away from tobacco, reinforcing the notion that producers with experience in growing other crops are most likely to be willing to experiment with new varieties. The majority of producers represented in our sample have diversified their crop portfolios, suggesting a level of willingness amongst Spanish and Italian growers to at least experiment with new cops.

### The Role of Producer Associations in the Transition to PMF

Our participants also had concerns about the implications of PMF for new agronomic practices and infrastructure. These concerns are important; the willingness of farmers to switch to biopharmaceutical tobacco has been shown to decrease as the need for new production methods and equipment increases – economic returns have to scale with these demands (Nevitt et al., 2003; Hayes et al., 2014). Nevitt et al. (2003) suggest that multi-season contracts would be required for tobacco farmers to invest in new equipment for PMF if required. However, our findings show that the demands of new production methods or equipment may be counterbalanced by the simplification of other processes, such as the suppression of flowering and/or the use of “green” rather than dried tobacco leaf (two processes that require significant time and money to manage). Other studies of European farmers showed a similar willingness to grow GM (food) crops providing that higher incomes and weed control were guaranteed (Areal et al., 2011); additional steps to ensure GM and non-GM crop co-existence policies were adhered to diminished this willingness, however. It is not clear whether the lack of tobacco wild relatives in Europe will lead to more lenience with respect to EU GM coexistence policies and distance-based planting for any potential future release of PMF plants (despite this being promoted as an advantage for certain PMF platforms). Spok and Karner (2008) note that even greater isolation ranges could be required for biopharmaceuticals due to the production of pharmaceutically active compounds, constituting a risk for the development of PMF in regions where smoking tobacco is grown.

Another risk for PMF is that the expected premiums for products do not compensate for the extra demands of these new tobacco varieties (including potential isolation distances) or required downstream processes. However, there is a concomitant opportunity for producer associations to help their members adopt new agricultural technologies. The importance of POs to the agricultural innovation processes has been somewhat overlooked (Menary et al., 2019), but our findings suggest that POs could play a vital role in the transition to PMF tobacco. In addition to providing their members with agronomic advice, contracts and loans of equipment and money, POs are trusted by their members and can also access EU rural development funding that single farmers cannot. The communal tobacco leaf-drying facilities in Extremadura were funded in such a way. The construction of contained facilities to grow PMF tobacco under license (in absence of more favorable EU regulation toward GM crops) could also be a possibility for these organizations. POs can also facilitate contractual agreements between growers and processors of PMF products, which suggests that those interested in taking PMF beyond contained facilities should first interface with such organizations.

Likewise, *Unitab*, a European-wide “association of associations” that lobbies EU institutions on behalf of its members and which was mentioned by several participants, could represent a higher-level intervention point for the promotion of PMF. There are a number of risks to this approach, however. The first is that these organizations may not perceive any value in new uses for tobacco – our own attempts to contact various POs had mixed success. The second risk is that the structure of the industry, with political and economic power concentrated in large, influential businesses, could make POs hesitant to support the development of new technologies seen to challenge the status quo. PMF already faces some challenges due to the technological “lock-in” of existing protein expression systems (e.g., prior legislation that favors cell culture technology and not plants and sunk investments in those systems) (Menary et al., 2020). Those companies explicitly opposed to genetic modification at this time – Imperial Brands (Our Views – Imperial Brands, 2019), British American Tobacco (British American Tobacco – Leaf research, 2019), and Altria Group (Altria Group, 2016) – may therefore perceive PMF as a contaminant threat to their non-GM crops, if not also a threat to the availability of growers themselves (Nevitt et al., 2003)^4^. This could pose a barrier to PMF at some point in the future. Despite claims to the contrary, we were unable to find tobacco-specific laws concerning genetic modification in the EU.

### Stakeholder Attitudes Toward PMF and NPBTs

Our findings describe (predominantly) favorable views toward PMF and NPBTs amongst our participants. These views appear to be driven in large part by the beneficial purpose of PMF, which outweighed the perceived risk of genetic modification for those few farmers who expressed concerns about GM food; some participants also assumed this would hold true for the general public. There is some evidence to suggest that the field of purpose is important for the social acceptability of plant biotechnology, health-related purposes being cited as more acceptable than ornamental purposes, for example (Connor and Siegrist, 2010).

Our findings support the hypothesis that third-generation biotechnology is more socially acceptable, at least for key stakeholders. It has also been suggested that whereas the benefits of first-generation biotechnology accrued to producers and seed companies, second- and third-generation GM crops offer distinct benefits to consumers and could change consumer opinion about biotechnology (Sakakibara and Saito, 2006; Moses and Goossens, 2017). These factors, combined with the potential for cisgenic or intragenic germlines, suggests that NPBT-bred tobacco for medical use is more likely to be socially acceptable than previous biotechnology applications.

Few environmental or ethical concerns were raised by participants with respect to NPBTs. As Thompson (2007) notes, these two factors have tended to be separated, environmental risk being treated primarily as a technical issue unrelated to ethics [though some authors have argued there is an environmental cost to not adopting biotechnology (Biden et al., 2018)]. Our participants did not cite either as significant areas of perceived risk.

### Reflections, Limitations, and Areas for Further Inquiry

Stilgoe et al. (2013) notes that scientists have political responsibility for new technology, as new technologies actively shape society. Our approach has been to take account of the potential synergies and obstacles with current tobacco production. These findings will be communicated with the Newcotiana consortium in order for scientists and others to understand how PMF can be responsibly scaled-up in Europe. However, a limitation of our approach is that the voices of other potential stakeholders have not been heard. Some people may resist any and all forms of genetic modification, such as organic farmers in Spain (Herrero et al., 2017). Although we did not limit contact to organizations with favorable views toward PMF or GMOs, groups with negative views may have excluded themselves from this study due to their reluctance to be associated with the technology (indeed, this was the reason cited by one tobacco producer association for avoiding involvement in the study). Another limitation is that due to the early stage of open-field tobacco development, important questions are unanswered: exact quantities of crop required, expected price for a given amount of tobacco and whether green or dried leaves (or a combination) are needed.

There is also a challenge in balancing the need to provide participants in such studies with enough information to ensure an informed debate, but without influencing participants by “over-framing” issues (Pidgeon et al., 2013). An area for further enquiry may be to more robustly assess participants’ knowledge of genetic modification techniques. If and when open-field PMF tobacco varieties become commercially available, a follow-up study – perhaps employing an action research approach – exploring how the technology is deployed and adapted would be of interest to agricultural technology adoption researchers.

## Conclusion

Given the stagnating production of and prices for tobacco in EU member states, the potential to incorporate PMF tobacco was seen as favorable by key stakeholders. The possibility for tobacco farming to become de-stigmatized – or at least, reduction of the stigma of tobacco production – with a new purpose for tobacco as a crop to produce important components for medicinal products, was also viewed favorably by current tobacco producers. There remain several unknowns: the attitudes of “big tobacco” toward PMF tobacco, as well as the need for additional infrastructure and potential profitability, were seen as potential risks to the scale-up of PMF tobacco. Our research suggests that close collaboration with these stakeholder groups – particularly POs, who have connections to farmers as well as purchasers – has the potential to identify and become an essential aspect for successful scale-up.

Importantly, we have demonstrated that there is demand for at least certain types of biotechnology amongst particular agricultural communities in the European Union. This may indicate an imbalance between current policy and stakeholder interest in these technologies.

## Data Availability Statement

The datasets generated for this study can be found in the Figshare depository: doi: 10.24376/rd.sgul.11841333.

## Ethics Statement

The studies involving human participants were reviewed and approved by the Saint George’s Research Ethics Committee. The patients/participants provided their written informed consent to participate in this study.

## Author Contributions

SF conceived of the study and acquired the funding. SF and JM designed the study. JM, MA, and AS undertook fieldwork and collected the data. JM, MH, AP, and SF carried out the data analysis. JM drafted the manuscript. All authors contributed to the final version.

## Conflict of Interest

The authors declare that the research was conducted in the absence of any commercial or financial relationships that could be construed as a potential conflict of interest.

## Footnotes

1 https://newcotiana.org

2 Technicians advise tobacco farmers on what plant varieties to use given the characteristics of their land, what plant protection products to use and agronomic advice. They are typically employed by POs.

3 Producers in the EU must belong to an association in order to grow tobacco.

4 Philip Morris International do not state a position toward genetic modification on their corporate website.
